# Integrated experimental and simulation study of the response to sequential treatment with erlotinib and gemcitabine in pancreatic cancer

**DOI:** 10.18632/oncotarget.7491

**Published:** 2016-02-19

**Authors:** Paolo Ubezio, Francesca Falcetta, Laura Carrassa, Monica Lupi

**Affiliations:** ^1^ Department of Oncology, IRCCS – Istituto di Ricerche Farmacologiche “Mario Negri”, Milano, Italy

**Keywords:** erlotinib, gemcitabine, pancreatic cancer, mathematical model, cell cycle

## Abstract

The combination of erlotinib with gemcitabine is one of the most promising therapies for advanced pancreatic cancer. Aiming at optimizing this combination, we analyzed in detail the response to sequential treatments with erlotinib → gemcitabine and gemcitabine → erlotinib with an 18 h interval, adopting a previously established experimental/computational approach to quantify the cytostatic and cytotoxic effects at G1, S and G2M checkpoints. This assessment was achieved by contemporary fits of flow cytometric and time-lapse experiments in two human pancreatic cancer cell lines (BxPC-3 and Capan-1) with a mathematical model reproducing the fluxes of cells through the cycle during and after treatment.

The S-phase checkpoint contributes in the response to erlotinib, suggesting that the G1 arrest may hamper S-phase cytotoxicity. The response to gemcitabine was driven by the dynamics of the progressive resumption from the S-phase arrest after drug washout. The effects induced by single drugs were used to simulate combined treatments, introducing changes when required. Gemcitabine → erlotinib was more than additive in both cell lines, strengthening the cytostatic effects on cells recovering from the arrest induced by gemcitabine. The interval in the erlotinib → gemcitabine sequence enabled to overcome the antagonist effect of G1 block on gemcitabine efficacy and improved the outcome in Capan-1 cells.

## INTRODUCTION

Pancreatic cancer is one of the leading causes of cancer death in the Western world and its lethality is principally ascribed to the fact that only 15–20% of patients are eligible for surgery and the best available therapies increase survival by only a few months [1]. Among cytotoxic drugs, gemcitabine was established as first-line standard chemotherapy but since its introduction in the late 1990s there have been no significant improvements in survival [2]. At present a combination of 5-fluorouracil, irinotecan and oxaliplatin or gemcitabine and nab-paclitaxel are considered standard treatments, having demonstrated significant improvement in median survival, global health status, and quality of life [3, 4].

Besides cytotoxic drugs, small molecules targeting the epidermal growth factor receptor (EGFR), such as erlotinib, have shown clinical activity as monotherapy or in combination. Simultaneous treatment with gemcitabine and erlotinib was also investigated in pancreatic cancer (reviewed in [5]), where EGFR is over-expressed in more than 50% of cases [6–8], and gave a significant but limited improvement in overall survival of approximately two weeks, so this regimen has not been widely adopted, in consideration of its added toxicity [2]. However, pre-clinical studies indicate that the order in which EGFR inhibitors and gemcitabine are given is important in determining a synergistic or antagonistic effect of the combination and simultaneous treatment may be antagonistic [9–11], suggesting there is room to improve the effectiveness of the therapy.

We believe that deeper knowledge, not only of the molecular processes but also of cell proliferation in response to treatment challenges, is vital to optimize the treatment schedules. The approach we propose has already been used to decode the dose- and time-responses to different anticancer agents [12–20]. It allows a detailed analysis of the dynamics of cell proliferation, reconstructing *in silico* the fluxes of the cells in the cycle, while interacting with the checkpoints in G_1_, S and G_2_M phases, and separating cytostatic from cytotoxic effects.

As the antiproliferative effect of the combination can depend on the different genetic background of the cells [21], we selected two human pancreatic cancer cell lines, BxPC-3 and Capan-1, both p53-mutated (point mutations A159V in Capan-1 and Y220C in BxPC-3) [22, 23] but differing in KRAS status (point mutation G12V in Capan-1 and wild type in BxPC-3) [22, 23] and EGFR protein expression levels (low in Capan-1 and high in BxPC-3) [24], and analyzed in detail the antiproliferative response to erlotinib and gemcitabine in both systems. The proliferation process was dynamically rendered *in silico* to interpret the response to combined treatments, providing a solid ground and new information for their evaluation.

## RESULTS

### Cell cycle effects of erlotinib and gemcitabine: experimental data

Before approaching the interpretation of combined treatments, we studied the complete time- and dose-dependence of the anti-proliferative cell cycle response induced by the single treatments in both cell lines. We collected flow cytometry (FC) and time-lapse (TL) data during and after treatment with different concentrations ranging from low-effective (about 30% growth inhibition) to high-effective (about 70% growth inhibition) according to preliminary growth inhibition experiments with Sulforhodamine B (SRB) assay.

**Figure 1:**
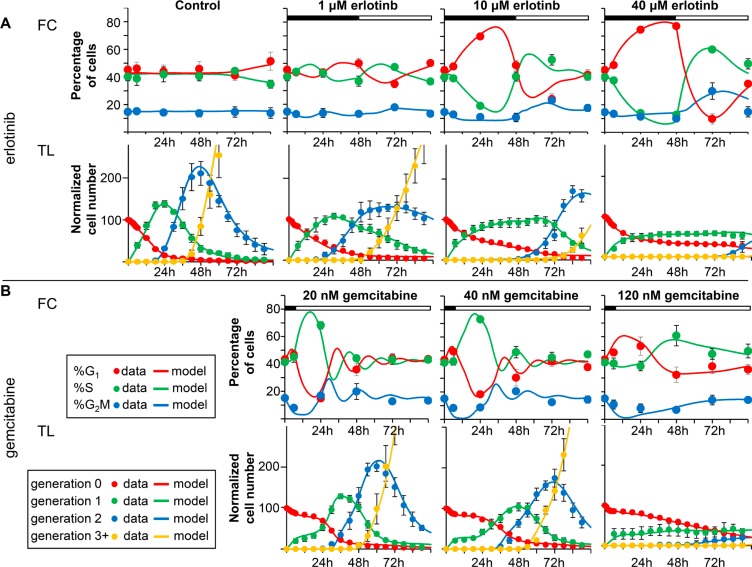
Experimental data and model prediction Data and fit with the model describing the effects of erlotinib (panel **A**) and gemcitabine (panel **B**) in BxPC-3 cells. Time courses of measurable quantities obtained from the final model (continuous lines) compared with experimental data (symbols), for each drug concentration. The good quality of the fit indicates that the model successfully predicts flow cytometry (FC) (%G_1_, %S, %G_2_M) and time-lapse (TL) data (number of cells in subsequent generations) for all doses. The symbols and error bars represent the mean and standard deviation of experimental data of at least three independent experiments (FC) or five replicate culture wells (TL).

Experimental data after treatments with erlotinib or gemcitabine on BxPC-3 are reported in Figure 1. Cell cycle distribution was only slightly altered by 1 μM erlotinib, with an increase of %G_1_ at the end of treatment (48 h) and a decrease at 72 h. The accumulation of cells in G_1_ was accentuated with 10 and 40 μM and already detectable at 24 h (Figure 1A–FC panels and Supplementary Figure 2). At 72 h cells were out of G_1_ and at 96 h the cell cycle was still altered only with 40 μM.

TL showed up the generation-dependence of the effects of erlotinib (Figure 1A – TL panels). In the first 6 h the curves representing the cells in generation 0 decreased similarly in control and treated samples, indicating that the cells that were in G_2_M at the beginning of the treatment could divide like the control. Afterwards the exit of the cells from generation 0 was dose-dependently delayed, and more than 20% were still undivided at 96 h in the samples treated with 40 μM erlotinib. In cells that were able to divide, cell cycle progression in generation 1 was again dose-dependently delayed, as demonstrated by their long stay in generation 1, their late appearance in generation 2 (after 48 h) in 10- and 40-μM treated samples (Figure 1A–TL panels), and the longer average cell cycle duration (Tc) (Supplementary Figure 3).

The anti-proliferative effects of erlotinib were confined to generations 0 and 1 and the cells were able to grow normally after two mitoses. Cell death was detected mainly among undivided cells treated with the highest concentrations (Supplementary Figure 3).

Figure 1B shows the results of 6 h treatment with gemcitabine. The main perturbation shown by FC was an increase of %S and a decrease of %G_1_ at 24 h in samples treated with 20 and 40 nM. DNA histograms (Supplementary Figure 4) indicated that a subpopulation of partially synchronized cells was propagating in S phase at that time. With 120 nM there was a lower, later increase of %S. An additional short-time effect was a decrease of %G_2_M, suggesting that cells treated while in G_2_M were able to reach G_1_, whereas entry in G_2_M was reduced. The short-time decrease of generation 0 cells confirmed this (Figure 1B – TL panels). After treatment, the number of cells in generation 0 remained stable up to 24 h, indicating a strong cytostatic effect even with 20 nM, then generation 0 cells divided and entered generation 1 at dose-dependent rates. Only cells treated with 120 nM were delayed in generation 1 and strong cell death was detected (Supplementary Figure 5).

The overall process of proliferation involves the passage of cell cohorts through the cell cycle and mitosis, while treatment changes the “unperturbed” cycling imposing delays or blocks and killing cells with different mechanisms in each phase. The complexity of the effects of erlotinib or gemcitabine was interpreted by rendering the complete cycling of the cell population through G_1_, S and G_2_M and in the subsequent generations *in silico*.

### Cell cycle effects of erlotinib and gemcitabine: simulation results

Core of the model are continuity equations which govern the dynamic evolution of the number of cells in each age and phase (see Supplementary Methods). The inputs of the model are parameters that describe the cell cycle during unperturbed growth (mean phase durations –${\bar{T}}_{G1}$, ${\bar{T}}_{S}$ and ${\bar{T}}_{G2M}$– with their variability, expressed as coefficients of variation – CV_G1_, CV_S_ and CV_G2M_) and the effects of treatment, altering the normal cell cycle flow. Each drug-induced effect is associated to a single parameter expressing its probability of occurrence. They include “delay” and “block” (cytostatic effects) and “death rates” (cytotoxic effects) in each cell cycle phase and generation. For instance, “block” is modeled assuming that when a cohort of coetaneous cells arrives at a given checkpoint the fraction of cells corresponding to the block probability is arrested, and the others go on and reach the next phase. In this way we can account for the fact that not all cells are arrested and we can “measure” the activity of the checkpoint – strong if the block probability is high, weak if it is low. Blocked cells may subsequently re-enter the cycle according to a “re-cycling rate”.

The researcher can build the model and simulate the proliferation at the desired level of complexity obtaining as output the time course of measurable quantities comparable with experimental data, like cell number and cell cycle percentages. Once the model of normal cycling of untreated cells was established by fitting control data, the model of treatments is obtained by optimizing the parameters of the perturbation modules in play.

The best-fit models of erlotinb and gemcitabine are represented in Figure 1, with the experimental data, showing that they closely predicted all FC and TL data with their time- and dose-dependence.

### Erlotinib

In order to simulate the effects induced by erlotinib we considered the two intervals 0–48 h (with the drug) and 48–96 h (after drug washout) separately. As erlotinib is supposed to prevalently affect G_1_ cells, we first attempted to fit FC and TL data with models including cell cycle perturbations only in G_1_ phase, but they were not able to provide reasonable fits, making predictions conflicting with either TL or FC data (not shown). To conciliate the delays observed in TL data with the time variations of cell cycle percentages obtained by FC, we needed to associate the G_1_ arrest with a delay in S phase and a mild G_2_M arrest.

**Figure 2:**
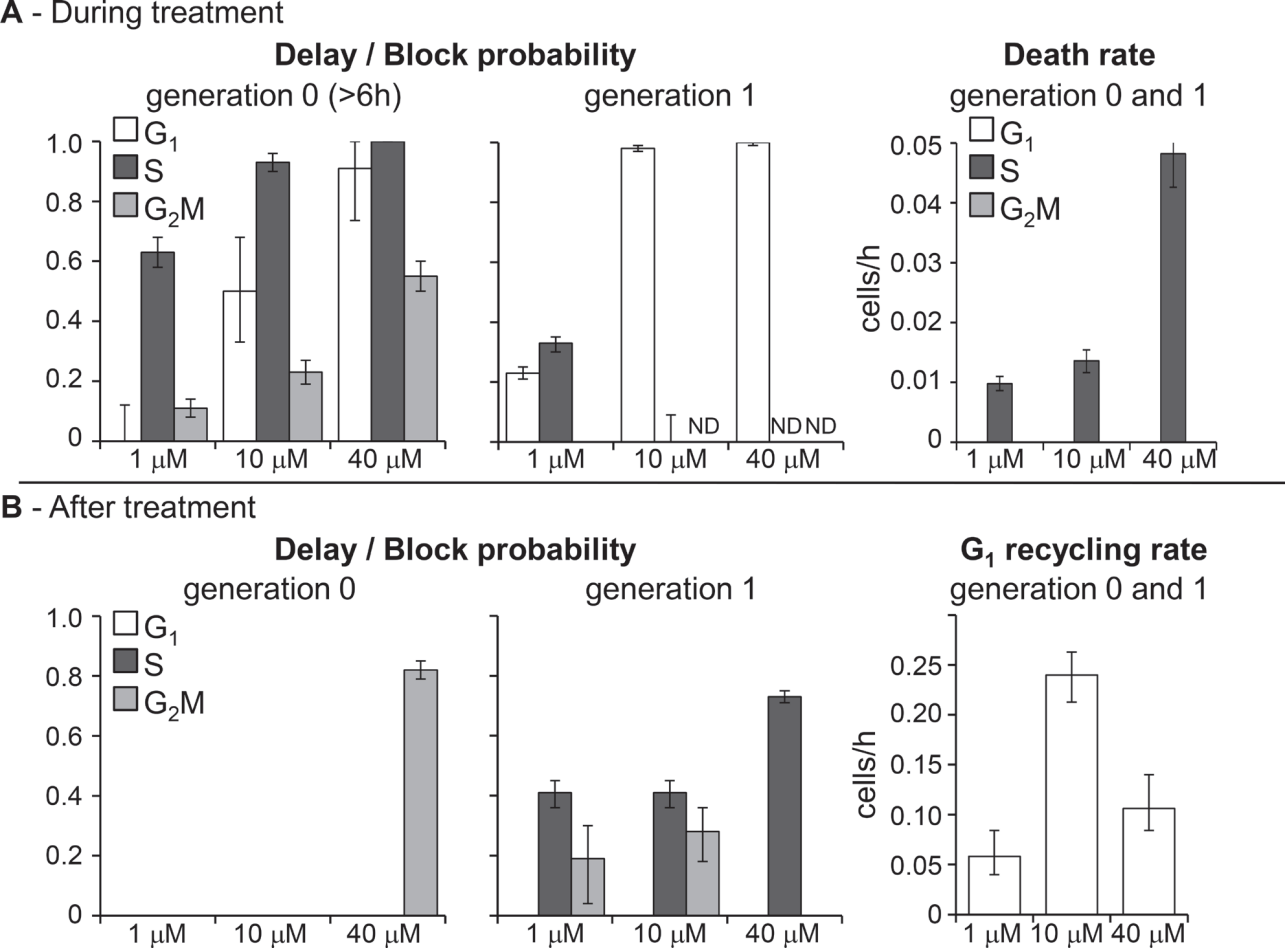
Parameters describing checkpoint activities in the best-fit model of erlotinib (**A**) Delay and block probability in G_1_, S and G_2_M during treatment (left panel). The effect was different for undivided cells (generation 0) and their descendants (generation 1). In generation 0, delay and blocking activity were time-dependent and reached the half-maximum 6 h after the beginning of treatment. In generation 1, block and delay were constant. Death rate of cells blocked in G_1_ or traversing S phase (right panel). (**B**) Cytostatic effects still present after treatment (left panel). No mortality was observed. Recycling rate of cells exiting G_1_ block was set equal in generation 0 and 1 and reached half-maximum at the end of treatment (48 h) (right panel). Error bars were calculated by applying the uncertainty analysis described in Supplementary Methods.

Figure 2 shows the dose-dependence of the main parameters of the erlotinib model, obtained fitting jointly the FC and TL time courses shown in Figure 1. Cellular response to erlotinib involved checkpoints in all phases, but they were not immediately activated. Data were fitted adopting a sigmoid time-dependence for generation 0 parameters describing delay and blocking activities, with half-maximum at 6 h. The dose-dependence was striking: G_1_ block appeared at 10 μM and was almost complete at 40 μM, S-phase delay was already strong (0.6 probability) with 1 μM, reached 0.9 with 10 μM and was almost complete at 40 μM, while a weak G_2_M block was detected at 10 μM and reached 0.5 probability at 40 μM (Figure 2A–generation 0). The delayed activation and the weakness of G_2_M checkpoint allowed cell divisions in the first hours of treatment, but newborn cells (generation 1) were then intercepted at G_1_ checkpoint and almost all of them were arrested when exposed to 10 μM or higher concentrations (Figure 2A–generation 1).

Cell death occurred in S (not in G_1_) phase in generation 0 with dose-dependent rate, but remaining low up to 10 μM.

After discontinuation of treatment (48 h), only the cells that had already reached generation 2 at 48 h continued to cycle without delays. Cells in generations 0 and 1 were no longer intercepted by G_1_ checkpoint and those previously blocked gradually re-entered the cycle (Figure 2B–G_1_ recycling rate), but progression through S and G_2_M phases was delayed (Figure 2B–delay/block probability). A high fraction of 40-μM treated cells was still in generation 0 after treatment and experienced a strong G_2_M delay, but cell death was not detected even with this high concentration.

### Gemcitabine

A model including delay and death only in S phase explained the trend of the observed data, but significantly better fits were obtained including a delay, without death, in G_1_ and further refinement led to include a modest, although dose-dependent, G_2_M block.

**Figure 3:**
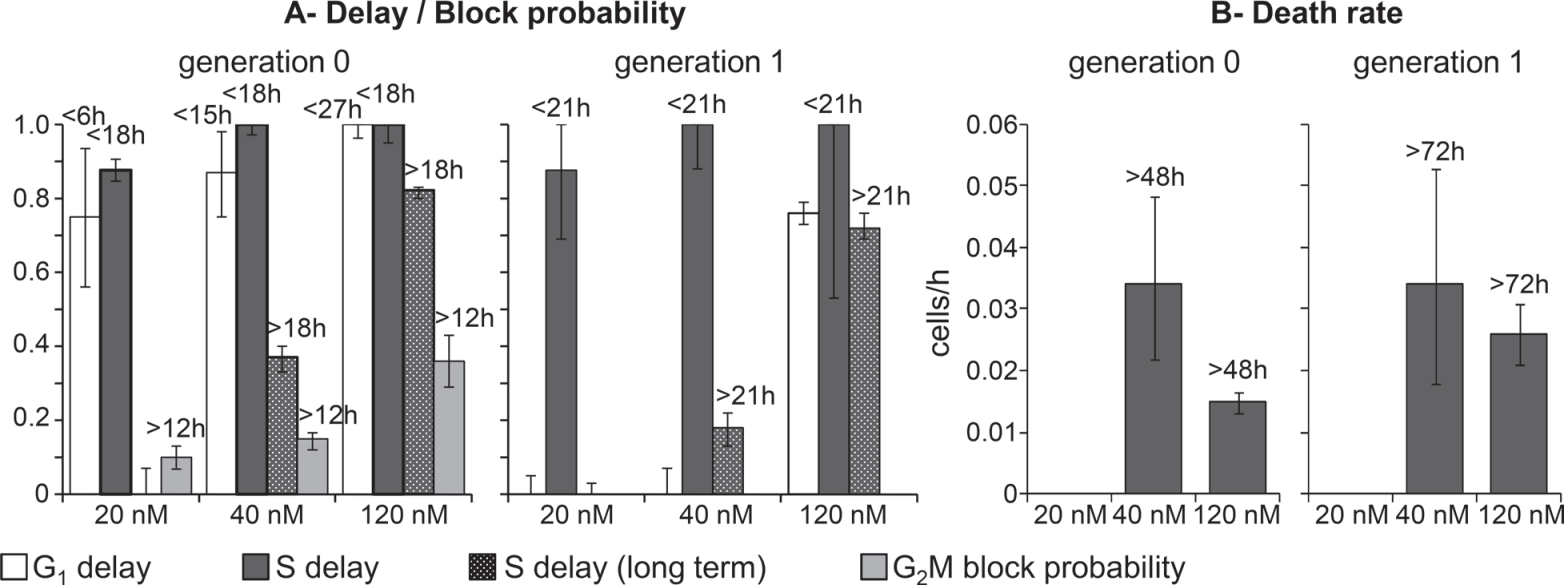
Parameters describing checkpoint activities in the best-fit model of gemcitabine (**A**) Dose-dependence of maximum delay and block probability in G_1_, S and G_2_M in generations 0 and 1. Long-term S phase delay was not zero with 40 nM and 120 nM. (**B**) Death rate of cells traversing S phase in generations 0 and 1. Time-dependent parameters reached half-maximum at the times reported over each column. Error bars were calculated as in Figure 2.

Gemcitabine immediately reduced the DNA synthesis rate (by 88% at 20 nM and with a complete inhibition at higher concentrations) maintaining the effect even after discontinuation of treatment. DNA synthesis gradually restarted, reaching about 50% of the normal rate at 18 h (Figure 3A – generation 0). This caused the formation of a wave of semi-synchronized cells, detected by FC analysis of DNA content (Supplementary Figure 4). According to the model, the timing of the restart of DNA synthesis was not dose-dependent, but recovery was incomplete at higher concentrations, maintaining a long term S delay. Cells treated with 20 nM that recovered from S-phase block eventually divided, and their descendants regularly cycled, while at higher concentrations, cells progressed slowly and those still in generation 0 at 48 h or in generation 1 at 72 h were committed to die (Figure 3B). The death rate with 120 nM was similar or lower than that with 40 nM, but the number of dead cells was much higher, because most cells were still in generations 0 and 1 when death occurred, while many 40-nM treated cells had already reached higher generations and survived.

In addition to S-phase related events, gemcitabine induced a strong G_1_ delay during treatment (generation 0). The delay was already active with 20 nM, and recovery times were dose-dependent, from 6 h (20 nM) to 27 h (120 nM) (Figure 3A). Only cells treated with 120 nM were still delayed in G_1_ after mitosis (generation 1). The mild perturbation of G_2_M phase did not involve cells that were in G_2_M during treatment, but only those in generation 0 that reached G_2_M later than 12 h and when the DNA synthesis inhibition was released.

### Effects on Capan-1 cells

**Figure 4:**
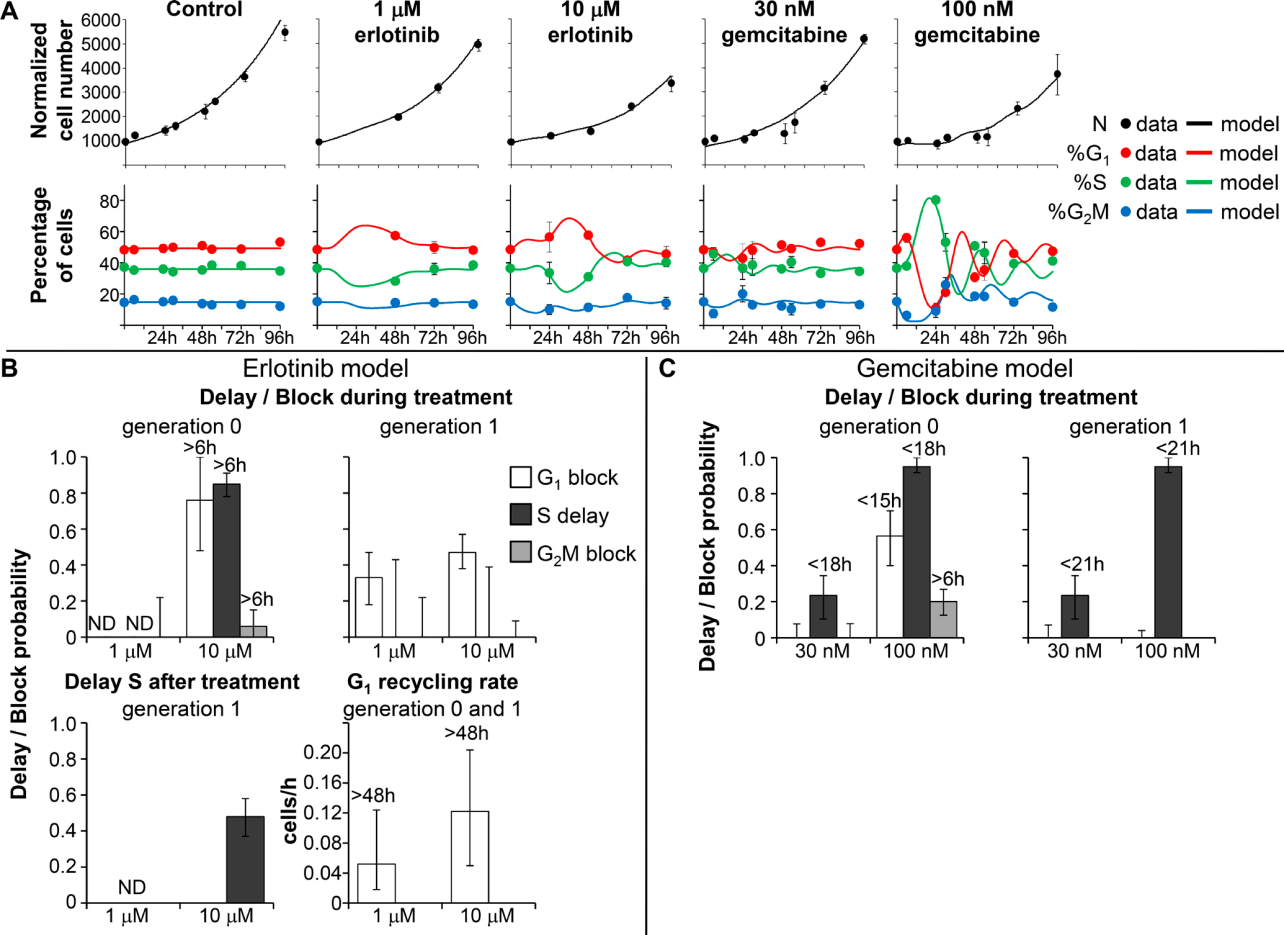
Experimental data and model prediction in Capan-1 cells (**A**) Time courses of cell number and flow cytometric percentages obtained from the final model (continuous lines) compared with experimental data (symbols) for untreated Capan-1 cells and samples exposed to erlotinib and gemcitabine. The symbols and error bars represent the mean and standard deviation of experimental data of three independent experiments. (**B**) Parameters describing checkpoint activities in the best-fit model of erlotinib. Delay and block probability in G_1_, S and G_2_M during treatment differed for undivided cells (generation 0) and their descendants (generation 1). Time-dependent parameters reached half-maximum at the times reported over each column. (**C**) Parameters describing checkpoint activities in the best-fit model of gemcitabine. Error bars in panels B and C were calculated by applying the uncertainty analysis described in Supplementary Methods.

The DNA distribution of Capan-1 cells during and after treatment with erlotinib and gemcitabine is shown in Figure 4A. The patterns of the response to treatments in Capan-1 were similar to those in BxPC-3, even though in Capan-1 higher drug concentrations were needed to induce the growth inhibition observed in BxPC-3. Flow cytometric analysis of Capan-1 cells exposed to 1 and 10 μM erlotinib indicated only a slight increase of %G_1_ during treatments. In treatments with gemcitabine, cells initially blocked in G_1_ and then progressing through S phase as a partially synchronized subpopulation were seen in samples exposed to 100 nM.

As Capan-1 cells could not be reliably tracked in TL movies because of their morphology and their piled-up, cluster-like spatial distribution, we partly bypassed the lack of TL data by fitting flow cytometric cell cycle percentages with the absolute cell number obtained with the Coulter counter at different times during and after treatment. We fitted both flow cytometric percentages and cell numbers (Figure 4A) with the model developed with the richer BxPC-3 dataset, considering the same kind of cell cycle perturbations and optimizing the parameters' values.

Capan-1 exposed to 1 μM erlotinib were able to divide regularly and the simplest model explaining the data required only a moderate block in G_1_ phase for cells in generation 1, with blocked cells exiting at the end of treatment and progressing regularly through the other phases (Figure 4B). In 10-μM treated cells, the perturbations were stronger in G_1_ and S phases, with reduced progression of the cells in generation 0, and only a very small percentage was intercepted by the G_2_M checkpoint. A fraction of the cells that were able to divide once (generation 1) were blocked in G_1_ and were progressively released after drug washout (see G_1_ recycling rate), then slightly delayed in S phase (Figure 4B). 10 μM erlotinib was only cytostatic, while some cell death was seen in BxPC-3.

The final models of Capan-1 treated with gemcitabine showed a 23% reduction of DNA synthesis rate in cells treated with 30 nM, and almost complete reduction with 100 nM (95%) (Figure 4C). In both samples, synthesis restarted after drug removal with kinetics similar to that of BxPC-3 cells, reaching about 50% of the normal value at 18 h. Similarly to BxPC-3, cells that were initially in G_2_M divided regularly and were delayed in generation 1. The G_1_ delay experienced by Capan-1 exposed to 100 nM was similar to that of BxPC-3 treated with 40 nM. The perturbation of G_2_M phase of cells that had recovered from the S-phase delay in generation 0 was confirmed as a secondary effect, present only at 100 nM. Cell death was not demonstrated by the modeling, suggesting that the effects of gemcitabine were prevalently cytostatic and not cytotoxic even at the highest concentration (Figure 4C).

### Combined treatments

As previous studies [9, 25] had demonstrated the superiority of sequential over simultaneous treatment with erlotinib and gemcitabine, we focused on two different sequences. In the first (G→E), 6 h incubation with gemcitabine was followed, after 18 h, by 48 h treatment with erlotinib, while in the second (E→G), the order of the two drugs was inverted, with the same interval.

Isobolograms of the two schedules and simultaneous 72 h treatment at different levels of efficacy (IC30 and IC50) are shown in Supplementary Figure 6. In BxPC-3 both schedules were close to additivity, with a synergistic trend in G→E (IC50) and an antagonist trend in E→G. In Capan-1 both sequences were synergistic, more at IC50 than at IC30. Simultaneous treatment led to higher CI in both cell lines, confirming the superiority of the sequential treatment.

The isobologram analysis provides a rough evaluation of the complexity of the response to treatment, nevertheless these results represented a first indication of the efficacy of the combined treatments that could be compared with other published studies. These preliminary experiments enabled to span the whole concentration-response range and were used to select the concentrations adopted in the subsequent detailed study.

To clarify the origin of the interaction suggested by the long-term SRB assay, we treated BxPC-3 and Capan-1 cells with low-effective concentrations of erlotinib (1 and 10 μM) and gemcitabine (20 and 40 nM for BxPC-3; 30 and 100 nM for Capan-1) in the two sequences. Cell count and DNA distributions (Supplementary Figures 7 and 8 – FC analysis) were collected for samples treated with single drugs or the combination at the end of the second treatment and 24 h later. Interpretation of these data required the support of modeling, because they are the result of the kinetics of checkpoint activation during the second treatment, intertwined with the history of each cohort of cells that flows through the cycle starting from different cell cycle positions as a consequence of the previous treatment.

### Analysis of combined treatment: results of the simulation

The models simulating single drug treatments, described above, were assumed to assess the expected effect of the combined treatment in the absence of drug interaction, and then compared with the data from the combination experiments.

### BxPC-3: G→E

At 24 h, when erlotinib was added, cells treated with gemcitabine presented a wave of semi-synchronized cells in S phase (see Figure 1B). The simulation ran with the gemcitabine single-treatment best model until erlotinib was added (24 h), then the cell cycle distribution reached at this time was applied as starting point for the erlotinib model.

**Figure 5:**
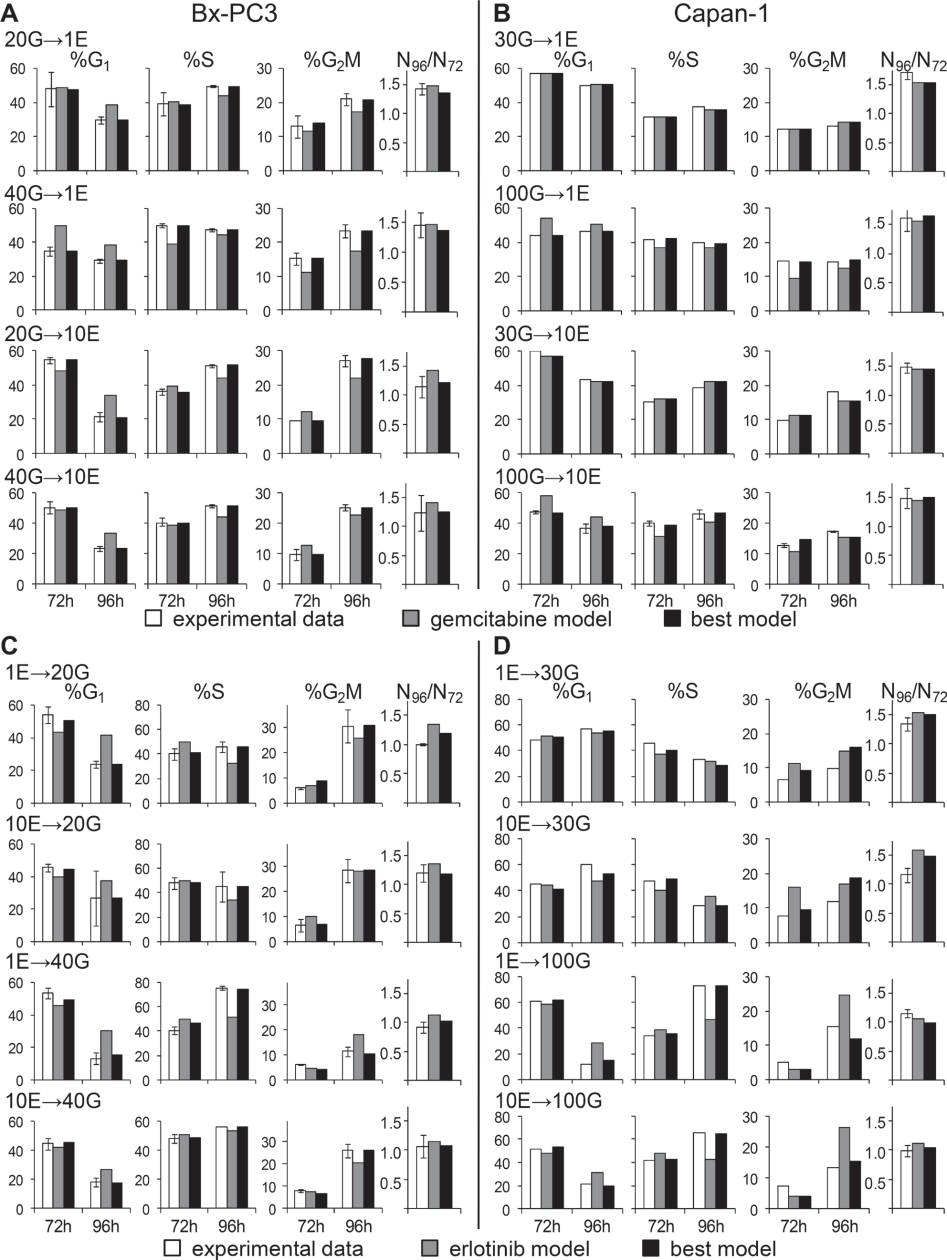
Experimental data from cell count and flow cytometric analysis after combined treatments compared with the best-fit model and the second drug model %G_1_, %S, %G_2_M and cell number increases between 72 h and 96 h were compared in BxPC-3 and Capan-1 after the G→E sequence (panels **A** and **B**) and after the E→G sequence (panels **C** and **D**).

Cell cycle percentages 48 h after the addition of erlotinib (72 h) and 24 h after its removal (96 h) were compared with those expected from the erlotinib model (Figure 5A). At 96 h the data indicated a lower %G_1_ and higher %G_2_M than with the erlotinib model. To reproduce these data we had to correct the erlotinib models as shown in Table 1A. Potentiation of the S and G_2_M cytostatic effects after treatment is the most probable source of a synergistic trend of this scheme, as was consistently predicted by the best models of all treatment groups, and is the only change required in the 40G→10E model. A reduction of the G_1_ block during the erlotinib treatment was suggested by the 40G→1E and 20G→10E best models, which on one hand reduces the cytostatic effect but on the other increases the number of cells entering S phase, where cell death occurred.

**Table 1:** Best-fit models for single treatment with erlotinib and G→E sequence

A								
	During treatment					After treatment		
**BxPC-3**	Delay S(> 6 h)	G_2_M blocking probability(> 6 h)	G_1_ blocking probability	Delay S	S deathrate(cells/h)	Delay S	Delay G_2_M	G_1_ recycling
	generation 0	generation 0	generation 1	generation 1	generation 0/1	generation 1	generation 1	generation 0/1
1 μMerlotinib	0.33 [0.30–0.35]	0.11 [0.08–0.14]	0.23 [0.21–0.25]	0.33 [0.30–0.35]	0.010 [0.008–0.011]	0.41 [0.36–0.45]	0.19 [0.04–0.30]	0.03 [0.02–0.04]
20G→1E	ND	0.11 [0–0.27]	0.23 [0.13–0.32]	0.15 [0–0.28]	0 [0–0.012]	**0.58** [0.51–0.65]	**0.59** [0.24–0.71]	0.07 [0.03–0.12]
40G→1E	ND	0.16 [0–0.31]	**0** [0–0.10]	0.27 [0.17–0.40]	0.016 [0.004–0.028]	**0.60** [0.53–0.66]	**0.58** [0.35–0.68]	ND
10 μMerlotinib	0.93 [0.91–0.96]	0.23 [0.19–0.27]	0.98 [0.97–0.996]	0 [0–0.09]	0.014 [0.012–0.016]	0.41 [0.39–0.44]	0.28 [0.18–0.36]	0.24 [0.21–0.26]
20G→10E	ND	0.07 [0–0.23]	**0.83** [0.70–0.91]	0 [0–0.19]	0.002 [0–0.020]	**0.56** [0.51–0.61]	**0.75** [0.63–0.82]	0.17 [0.13–0.21]
40G→10E	ND	0.10 [0–0.23]	0.91 [0.74–1.0]	0 [0–0.28]	0.018 [0.002–0.034]	**0.55** [0.50–0.60]	**0.59** [0.42–0.69]	0.23 [0.20–0.24]

B							
	During treatment				After treatment			
**Capan-1**	Delay S	G_2_M blocking probability(> 6 h)	G_1_ blocking probability	Delay S	S deathrate(cells/h)	Delay S	G_1_ recycling
	generation 0	generation 0	generation 1	generation 1	generation 0	generation 1	generation 0/1
1 μMerlotinib	ND	0 [0–0.39]	0.33 [0.18–0.47]	0 [0–0.43]	0 [0–0.018]	ND	0.03 [0.01–0.06]
30G→1E	ND	0 [0–0.18]	0.33 [0.21–0.49]	ND	0 [0–0.037]	ND	0.03 [0.01–0.07]
100G→1E	**0.78 (> 0 h)** [0.70–0.82]	0 [0–0.17]	**0** [0–0.13]	ND	0 [0–0.008]	ND	ND
10 μMerlotinib	0.85 (> 6 h) [0.78–0.91]	0.06 [0–0.15]	0.47 [0.38–0.57]	0 [0–0.39]	0 [0–0.004]	0.48 [0.37–0.58]	0.06 [0.03–0.10]
30G→10E	ND	0.06 [0–0.22]	0.47 [0.37–0.65]	0.48 [0.19–0.54]	0 [0–0.017]	0.48 [0.19–0.54]	0.06 [0.01–0.10]
100G→10E	**0.81 (> 0 h)** [0.69–0.90]	0.06 [0–0.19]	0.29 [0.10–0.47]	0.45 [0.30–0.56]	0 [0–0.004]	0.45 [0.30–0.56]	0.06 [0.01–0.19]

Shown in bold characters are the parameters describing the effects of the last administered drug that have to be changed to reproduce the experimental data of the combinations. The likelihood-based 95% confidence intervals for each parameter are in square bracket.

ND: not detectable, i.e. data were not sensitive for evaluation of the parameter. Short term G_1_ block was always ND with the combination (not shown). Parameters of erlotinib or the combination G→E were compared for BxPC-3 (panel A) and Capan-1 (panel B).

### Capan-1: G→E

Data for the combinations 30G→1E and 30G→10E were reasonably fitted with the erlotinib model without correction, but modifications were required for samples pre-treated with 100 nM (Figure 5B). The best models of both 100G→1E and 100G→10E (Table 1B) suggested that the G_1_ block was weakened or abolished and the DNA synthesis was strongly inhibited immediately after addition of erlotinib, without the 6 h lag observed with single treatment. The modest residual long term S-phase delay caused by gemcitabine was strengthened by the second drug. Thus potentiation of the effects against the cells traversing S phase may lead to synergism of the combination, with a mechanism acting in S phase before their division.

### BxPC-3: E→G

As shown in Figure 5C, the reference gemcitabine models were unable to predict experimental data properly, requiring refinement of the parameters in all phases (Table 2A). Generation 0 cells showed a trend toward a stronger G_1_ delay in the short term, confirmed in all treatment groups, and increased delays/block in S and G_2_M in the long term, which may counteract the cell death expected after removal of gemcitabine.

**Table 2:** Best-fit models for single treatment with gemcitabine and E→G sequence

A					
**BxPC–3**	Delay G_1_	Delay S	Long termDelay S (>18 h)	G_2_M blockingprob. (>12 h)	Long termDelay S (>21 h)
	generation 0	generation 0/1	generation 0	generation 0	generation 1
20 nMgemcitabine	0.75 (< 6 h) [0.56–0.93]	0.88 [0.85–0.91]	0 [0–0.07]	0.10 [0.07–0.13]	0 [0–0.03]
1E→20G	1 (< 6 h) [0.83–1]	0.68 [0.58–0.77]	**0.54** [0.39–0.68]	**0.60** [0.41–0.78]	**0.23** [0.09–0.37]
10E→20G	1 (< 6 h) [0.74–1]	0.95 [0.81–1]	**0.23** [0.11–0.33]	0.26 [0.10–0.43]	0.16 [0.01–0.48]
40 nMgemcitabine	0.87 (< 15 h) [0.75–0.98]	1 [0.97–1]	0.37 [0.33–0.40]	0.15 [0.12–0.17]	0.18 [0.13–0.22]
1E→40G	1 (< 15 h) [0.89–1]	0.96 [0.88–1]	**0.96** [0.89–1]	ND	0.04 [0–0.23]
10E→40G	0.98 (< 15 h) [0.68–1]	0.97 [0.90–1]	**0.52** [0.41–0.62]	**0.48** [0.24–0.71]	0.07 [0–0.26]

B					
**Capan–1**	Delay G_1_	Delay S	Long termDelay S (> 18 h)	G_2_M blockingprob. (> 6 h)	Long termDelay S (> 21 h)
	generation 0	generation 0/1	generation 0	generation 0	generation 1
30 nMgemcitabine	0 [0–0.08]	0.23 [0.10–0.34]	0 [0–0.71]	0 [0–0.08]	0 [0–0.32]
1E→30G	0 [0–0.07]	0.39 [0.24–0.55]	0 [0–0.33]	0 [0–0.05]	0 [0–0.55]
10E→30G	0 [0–0.26]	**0.59** [0.48–0.70]	0 [0–0.09]	0 [0–0.04]	0 [0–0.25]
100 nMgemcitabine	0.57 (< 15 h) [0.40–0.70]	0.95 [0.92–1]	0.29 [0.24–0.34]	0.20 [0.13–0.27]	0.10 [0.01–0.20]
1E→100G	0.74 (< 15 h) [0.52–0.96]	0.95 [0.84–0.99]	**0.82** [0.71–0.94]	ND	0 [0–0.12]
10E→100G	0.90 (< 15 h) [0.63–0.99]	0.95 [0.84–99]	**0.64** [0.56–0.73]	0.20 [0–0.40]	ND

Parameters of gemcitabine or the combination E→G were compared for BxPC-3 (panel A) and Capan-1 (panel B).

### Capan-1: E→G

Experimental data from Capan-1 cells were reasonably reproduced by strengthening the delay in G_1_ (100 nM gemcitabine only) and S phase (Figure 5D and Table 2B). Thus, in a scenario where cytotoxic effects were almost negligible, erlotinib pre-treatment strongly hampered the resumption of cycle progression after gemcitabine.

### Western blot analysis in drug combination schedules

**Figure 6:**
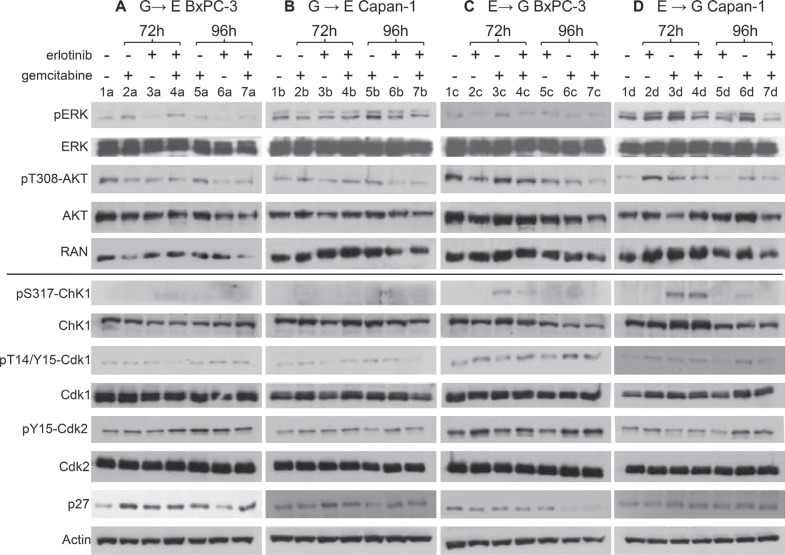
Effects of single treatments or combinations on proteins involved in the downstream signaling of the EGFR pathway and on cell cycle checkpoint-related proteins Western blot analysis showing protein levels in BxPC-3 and Capan-1 cells treated with G→E (panels **A** and **B**) and E→G (panel **C** and **D**). In all experimental groups, cells were treated with the single drugs (10 μM erlotinib, respectively 40 and 100 nM gemcitabine for BxPC-3 and Capan-1) or the combination and protein extracts were taken at the end of the last treatment (72 h) and 24 h after drug washout (96 h). Single treated samples were incubated with the drug at the same times scheduled for the combination (0–6 h for gemcitabine in the G→E group and 66–72 h in the E→G group; 24–72 h for erlotinib in the G→E group and 0–48 h in the E→G group). RAN and actin were used as loading controls.

The pathways involved in cell responses to the single and combined treatments were investigated by western blot analysis at the end of the combined treatment (72 h) and 24 h later (96 h), with the highest drug concentrations used in the combination studies (Figure 6).

Despite the different genetic backgrounds of the two cell lines, some common hallmarks could be indentified in the effects on the proteins involved in the downstream signaling of the EGFR pathway.

Phosphorylation of ERK and AKT showed opposite constitutive levels in BxPC-3 (high pT308-AKT and low pERK, lanes 1a, 1c) and Capan-1 (low pT308-AKT and high pERK, lanes 1b, 1d). In BxPC-3, single erlotinib treatment caused reduction of both pERK and pT308-AKT at the end of exposure (3a) and 24 h later (6a) while in Capan-1 their constitutive levels remained (3b, 6b). In BxPC-3 the G→E combination maintained low levels of pT308-AKT but pERK was not reduced (4a, 7a), while in Capan-1 the response to erlotinib was not affected by gemcitabine pre-treatment (4b, 7b).

In Capan-1 pERK and pT308-AKT rose immediately at the end of gemcitabine treatment (3d), whereas in BxPC-3 AKT phosphorylation did not increase (3c) and pERK increased only long time after treatment (2a). Erlotinib pre-treatment reduced the levels of pERK and pT308-AKT only in Capan-1 (4d, 7d); in BxPC-3 it did not change the status of pERK and pT308-AKT determined by gemcitabine (4c and 7c).

Western blot analysis of cell cycle checkpoint-related proteins showed only a few differences in the effects of the combinations compared to the single drugs. Chk1 was activated immediately at the end of gemcitabine (72 h in the schedule E→G) in both cell lines (pS317-Chk1 3c, 3d), followed by a decrease at 24 h, parallel with the strong inhibition of DNA synthesis and its recovery. In BxPC-3, pS317-Chk1 was reduced by erlotinib pre-treatment (4c) but remained highly present in Capan-1 (4d). At 96 h, phosphorylation in both cell lines treated with E→G was undetectable (7c, 7d).

Erlotinib did not activate Chk1 in either cell line (pT14/Y15-Cdk1 3a, 3b and 6a, 6b), while high p27 levels were detected at the end of drug exposure (3a, 3b), falling back to basal values 24 h later (6a, 6b), parallel to the presence of a G_1_ block and its release after drug removal. In BxPC-3, the G→E combination maintained the high expression of p27, pT14/Y15-Cdk1 and pY15-Cdk2 at 96 h (7a), while in Capan-1 the response to erlotinib was not substantially modified by gemcitabine pre-treatment (4b, 7b).

p27 was up-regulated in BxPC-3 long after gemcitabine (2a, 5a) in the absence of any G_1_ block but in presence of cell death. This effect might also explain the long-term over-expression of p27 in the G→E group (7a). In Capan-1 cells, where cell death was negligible, we did not detect any p27 upregulation (2b, 5b).

No effects of gemcitabine on Cdk1 phosphorylation were immediately detectable at the end of treatment (3c, 3d), but pT14/Y15-Cdk1 levels were high 24 h later in both cell lines (6c, 6d). This was in agreement with the progression in G_2_M at this time of the wave of semi-synchronized cells originating in S phase. pT14/Y15-Cdk1 expression was reduced in the E→G group. As regards G_1_/S-related proteins, gemcitabine and E→G treatment increased pY15-Cdk2 at 96 h in both cell lines (6c, 6d and 7c, 7d), coherently with the presence of G_1_ and S delay.

## DISCUSSION

Although EGFR signaling inhibition strategies have been investigated for years, studies on cell cycle effects of these compounds alone or in combinations have not yet analyzed the time- and dose-dependence of their cytostatic and cytotoxic effects and the origin of the potentiation with a combination.

The cell response to a drug comprises a complex sequence of events superimposed on a widely heterogeneous environment where some cells die, some do not proliferate at all, and some divide once or more during the observation period. This heterogeneity explains, at least in part, the failure of early attempts to optimize treatment schedules based on cell kinetics or synchronization. Alternative rationales have been proposed to design more efficacious treatments, for instance testing drug combinations able to preserve the population of normal cells [26–28]. For this aim, pretreatments with low doses of kinase inhibitors were investigated, as they can arrest the growth in normal cells, protecting them from subsequent chemotherapy, and contemporaneously sensitize cycling cancer cells to apoptosis induced by chemotherapy [29–32]. However, to exploit in practice theoretically sound rationales of combined treatments, it is essential to understand the antiproliferative effects of a second drug on cells which had received a first insults, e.g. with a compound silencing a specific target, and are not completely recovered.

We look into the complexity of the antiproliferative response to treatment using different techniques (principally FC and TL microscopy) and decoding the components of the response by simulation. This experimental/computational approach, able to quantify the activity of the checkpoints involved in the treatment, has been already applied in studies of the effects induced by cisplatin [13], taxol [14], topotecan [15], doxorubicin [17], melphalan [16], trabectedin [33] and radiation [20].

Here we apply the same method to study sequential treatments with erlotinib and gemcitabine in two pancreatic cancer cell lines (BxPC-3 and Capan-1) with different genetic backgrounds. This combination has been already considered in clinical studies for treatment of pancreatic cancer, however it is the first time that a full disclosure of the cell cycling during and after treatment with these drugs, singly and in sequence, is reported, illustrating the dynamics of the activity of cell cycle checkpoints.

Working in the range of concentrations corresponding to those observed in the plasma of patients treated with erlotinib (1–10 μM) [34], we found an important role for the S-phase checkpoint, not recognized in previous studies of cell cycle perturbations induced by EGFR inhibitors [35]. In BxPC-3 cells, erlotinib up to 10 μM had a prevalently cytostatic effect. Cytotoxic effects prevailed at higher concentrations, with cells dying in S phase. Thus the G_1_ checkpoint did not drive the cells towards apoptosis but acted as a protective mechanism, preventing cells reaching the more sensitive S phase where erlotinib exerted its cytotoxic effects.

The gemcitabine model indicated that the kinetics of cell responses was dominated by the timing of the S-phase checkpoint, which remained active several hours after the time of drug exposure. Full recovery of the DNA replication rate was achieved only at low gemcitabine concentrations, whereas in samples exposed to more cytotoxic doses the cells that did not complete generation 1 at 72 h eventually died in S phase. The same models of response to treatment were successfully applied to Capan-1 cells, even though at higher concentrations than those used in BxPC-3.

The time- and dose-dependence of the effects of erlotinib and gemcitabine singly was used to interpret their interactions in sequential treatments (G→E and E→G with 18 h interval). Cells were hit by the second treatment while recovering from the effects of the first, which were not yet over.

The positive interaction of the G→E scheme originated from potentiation of the cytostatic effects in S phase, with an immediate strong delay in Capan-1 and potentiation of S and G_2_M delays after the end of erlotinib in BxPC-3. This sequence was particularly favorable when a weakly effective erlotinib concentration followed a gemcitabine concentration causing an important S-phase arrest (like in 100G→1E sequence in Capan-1).

In BxPC-3 the E→G sequence caused an imbalance in the response to gemcitabine in cell cycle phases, strengthening G_1_ block and re-modulating the S-phase arrest, weaker in the short term but longer and associated with a G_2_M block. However, the added value of erlotinib pre-treatment on G_1_ and S short-term cytostatic effects was modest, also considering that at these times erlotinib induced an S delay that obviously could not boost the almost complete S-phase arrest induced by gemcitabine. The long-term G_2_M block may in part counteract the cytotoxic effects of gemcitabine. The balance of these effects is in keeping with the nearly additive effect in the SRB test. In Capan-1 the S-phase short-term delay was strengthened with 30 nM gemcitabine, while combinations with higher concentrations greatly increased the G_1_ delay in the short term and the residual S delay in the long term.

Knowing that the effect of the erlotinib and gemcitabine combination is a consequence of both cell cycle and growth factor signaling interactions [11, 36], we investigated some molecular pathways affected by these two drugs, especially those that might have an important role in the combination. In agreement with the literature [10, 37, 38], erlotinib and gemcitabine influenced the activity of the KRAS/ERK and PI3K/AKT pathways, though in different ways. In KRAS wt BxPC-3 cells, EGFR inhibitors inactivated ERK, while pERK had to be increased by gemcitabine for apoptosis [37]. Erlotinib's ability to inactivate ERK in BxPC-3 was partially reversed by the G→E combination and might be an important determinant of synergism [21].

This pathway was regulated differently in cells harboring the KRAS mutation (Capan-1) but, according to Bartholomeusz et al. [10], in this case the molecular mechanism behind the synergism might be a decrease in pT308-AKT expression, clearly observed at 72 h in Capan-1 treated with the E→G sequence. Other effects on the expression of cell cycle-related proteins can be interpreted as a consequence of cell cycle redistribution and the effects on KRAS/ERK and PI3K/AKT pathways.

Generally, the G→E sequence was preferable, since it was synergistic/additive in both cell lines, although there was no frank antagonism even with the opposite schedule. This might be a result of the 18 h interval between the two treatments, that enabled most G_1_ cells arrested by erlotinib to exit the block, avoiding the mechanism of antagonism reported for the simultaneous treatment, according to which G_1_ blocked cells are poorly sensitive to gemcitabine [11, 39].

As concerns the interval in the G→E sequence our analysis indicated that the timing of recycling from G_1_/S phase arrest did not depend on the gemcitabine concentration; it was the same in both cell lines and cells exiting from the block took several hours to complete S phase. That means that the optimal interval between treatments may range from 18 h to 24 h or more [40], making this rationale possible for *in vivo* translation. We also found that low, modestly effective concentrations such as 1 μM were enough to enhance gemcitabine's effect. However, this is not guaranteed in all patients inside a poorly vascularised pancreatic carcinoma with desmoplastic stroma, despite plasma levels above 10 μM. Insufficient drug delivery to the tumor site probably contributed to the statistical lack of improvement observed in a recent trial where a G→E sequence with one day interval was studied [41]. The trial was suspended due to lack of funding after recruitment of only 30 patients but no unexpected toxicity was observed. We believe that with ongoing improvements in drug delivery, targeting the microenvironment or using new drug carriers [42, 43], the efficacy of treatments in general and of the G→E scheme in particular could be boosted.

## MATERIALS AND METHODS

### Cell culture and drug treatment

Capan-1 and BxPC-3 cells were maintained as monolayers in T-25 cm^2^ tissue culture flasks (Iwaki) according to ATCC instructions. Culture was maintained in an incubator with 5% CO_2_ in air, at 37°C and 96% relative humidity. Exponentially growing cells were treated with erlotinib (Selleckchem) and/or gemcitabine (Lilly). After treatment the cells were washed twice with warm PBS and left in drug-free medium. At each time cells from three replicated flasks were detached, using 1 mL 0.05% trypsin-0.02% EDTA (Cambrex) in PBS, counted with a Coulter Counter ZM (Coulter Electronics), then pooled and fixed in cold 70% ethanol.

### Flow cytometry

Cells were harvested and prepared for flow cytometry as previously described [15]. DNA histograms were analyzed as described [44].

### Time-lapse

Cells were seeded in a multi-well plate, 24 h later the plate was placed on a time-lapse instrument designed to capture phase contrast images (Imaging Station Cell^R, Olympus). Sequences were captured every 20 minutes for 96 h, for one field for each well. We analyzed five replicate wells for each experimental condition [20]. BxPC-3 cells were followed up to 96 h or four generations, but Capan-1 lineage in TL movies could be reliably tracked only in controls up to the first division because of their morphology and their piled-up, cluster-like distribution.

### Analysis of combination treatment

To evaluate the effect of the combination of erlotinib and gemcitabine we used a standard growth inhibition assay with Sulforhodamine B detection. Cells were seeded in 96-well plates, treated 24 h later and after drug washout were left in drug-free medium up to seven days [40]. Using a factorial experimental design, each schedule was tested in at least two independent experiments, each with four independent replicate plates.

Data were analyzed using the isobologram method [45]. Cells were exposed to a range of doses of both drugs and combined concentrations inducing 30% and 50% growth inhibition were calculated by fitting dose–response curves of erlotinib at each gemcitabine concentration and vice versa. To quantify the interaction, we calculated the Combination Index (CI) for each pair of drug concentrations tested, according to the Lowe additivity criterion [46].

### Western blot analysis

Proteins were extracted and visualized using standard techniques as already described [47]. Specifically, primary anti Chk1 (G4), pT14/Y15-Cdk1, Cdk1, Cdk2, pERK, ERK, p27 and actin were purchased from Santa Cruz Biotechnology. Primary anti pS317-Chk1, pT308-AKT and AKT were purchased from Cell Signaling Technology. Primary anti pY15-Cdk2 was purchased from Abcam. The mouse monoclonal anti-Ran (clone 20) is from BD Transduction Laboratories.

## SUPPLEMENTARY MATERIALS FIGURES


