# Supplementary material for: Integrated experimental and simulation study of the response to sequential treatment with erlotinib and gemcitabine in pancreatic cancer
Source: Oncotarget. 2016 Feb 19;7(13):15492–506. doi: 10.18632/oncotarget.7491 (PMC4941256; doi:10.18632/oncotarget.7491)
Supplement: Supplementary file 1 [file oncotarget-07-15492-s001.pdf]

Integrated experimental and simulation study of the response to sequential treatment with erlotinib and gemcitabine in pancreatic cancer

Supplementary Materials

Computer simulation of cell cycle and drug effects

A thorough description and discussion of the approach has been published before [1–5], here we will sketch the outlines of the modeling.

First we modeled the basic cell proliferation process in untreated cells (controls) and then we modeled proliferation during and after treatment applying perturbations to basic cell cycling to reproduce the data of time course experiments with different drug concentrations and schedules.

Basic cell cycle model (untreated cells)

Wishing to consider not only the overall cell number, as in traditional pharmacodynamic models, but also the amount of information conveyed by the time courses of flow cytometry and time-lapse experiments, we introduced a proper cell cycle and age structure. The basic dynamics of progression inside each phase is described by continuity equations, in discrete time intervals Δ (from 0, i.e. the start of the experiment, to t_{end} at the end of the experiment) on the age distributions of cells in each phase. The "state vectors" $N_{ph}(a_i,t)$ represent the number of cells at age a_i in a phase ph (G_1, S or G_2M) at time t. Each phase is divided in k_{ph} compartments ($k_{ph}\Delta = T_{ph}$, where T_{ph} is the duration of phase *ph*) grouping cells with ages respectively from 0 to Δ (a_1), from Δ to 2Δ (a_2)… from $(k_{ph}-1)\Delta$ to $k_{ph}\Delta$ (a_{kph}). Model parameters are the durations of cell cycle phases (T_{G1}, T_S, T_{G2M}). Thus the time evolution of $N_{ph}(a,t)$ within a phase is given by:

$$N_{ph}(a_{i+1}, t+\Delta) = N_{ph}(a_i, t)$$

while cells entering a phase are those at the end of the previous phase at the previous time step:

$$N_{G1}(a_1, t+\Delta) = 2\, N_{G2M}(a_{kG2M}, t)$$
$$N_S(a_1, t+\Delta) = N_{G1}(a_{kG1}, t)$$
$$N_{G2M}(a_1, t+\Delta) = N_S(a_{kS}, t)$$

where the factor 2 in the first equation accounts for the fact that two daughter cells are produced for each mitotic cell ending the previous cycle.

The overall number of cells is obviously $N(t) = \Sigma_{ph}\Sigma_i N_{ph}(a_i,t)$ while typical flow cytometric data like percentages of cells in cell cycle phases (%G_1, %S and %G_2M) can be easily derived, e.g. $\%G_1(t) = 100 \times \Sigma_{aG1} N_{G1}(a_{G1},t)/N(t)$.

When dealing with time-lapse data, a further generation structure can be trivially included, repeating the previous equations in each cell generation (gen_h, starting from gen_0 of the cells at the start of the experiment):

$$N_{ph,genh}(a_{i+1}, t+\Delta) = N_{ph,genh}(a_i, t)$$
$$N_{G1,genh}(a_1, t+\Delta) = 2\, N_{G2M,gen(h-1)}(a_{kG2M}, t)$$
$$N_{S,genh}(a_1, t+\Delta) = N_{G1,genh}(a_{kG1}, t)$$
$$N_{G2M,genh}(a_1, t+\Delta) = N_{S,genh}(a_{kS}, t)$$

This structure enables to derive the overall cell number in each generation, and compare modeling prediction with the corresponding experimental results obtained by cell tracking in time-lapse experiments.

However this first basic model with fixed phase durations is not realistic and would be inconsistent with the direct time-lapse measure of intermitotic times, demonstrating a wide distribution of values among individual cells also in a homogenous environment, as it occurs in culture flasks in *in vitro* studies. In the cell lines used in the present work, intermitotic times varied from 12 h to 48 h (Supplementary Figure 1) with typical right-skewed distributions, well described by a log-normal or, better, by a reciprocal-normal function [5, 6]. Thus, at the second level of complexity of the model, intercell variability was included, assuming that the duration of each phase is distributed according to a reciprocal-normal ($F_{ph}(a)$), specified by its mean ($\bar{T}_{ph}$) and standard deviation or coefficient of variation (CV_{ph}). The distribution of intermitotic times F(Tc) is calculated as

$F(Tc) = \sum F_{G1}(a_{G1})\ F(a_S)\ F(a_{G2M})$ where the summation includes all combinations of a_{G1},a_S, aG_{2M} such that $a_{G1} + a_S + a_{G2M} = Tc$

F_{ph} enables to calculate the probability that cells having reached the age a_i will complete and exit the phase *ph* in the following time step as:

$$\beta_{ph}(a_i) = \frac{F_{ph}(a_i)}{1 - \sum_{j=1}^{i-1} F_{ph}(a_j)}$$

We obtain a model with six parameters: $\bar{T}_{G1}$, CV_{G1}, $\bar{T}_S$, CV_S, $\bar{T}_{G2M}$, CV_{G2M}, from which the coefficients $\beta_{ph}(a_i)$ are calculated and the dynamics reconstructed step by step

with the following balance equations, connecting the cell cycle distribution at time t with that at t+Δ:

$$N_{ph,genh}(a_{i+1}, t+\Delta) = (1-\beta_{ph}(a_i))\, N_{ph,genh}(a_i,t)$$

$$N_{G1,genh}(a_1, t+\Delta) = 2\, \Sigma_{am}\, \beta_{G2M}(a_m)\, N_{G2M,gen(h-1)}(a_m,t)$$

$$N_{S,genh}(a_1, t+\Delta) = \Sigma_{am}\, \beta_{G1}(a_m)\, N_{G1,genh}(a_m,t)$$

$$N_{G2M,genh}(a_1, t+\Delta) = \Sigma_{am}\, \beta_{S}(a_m)\, N_{S,genh}(a_m,t)$$

Intercell variability (if constant) drives the cell population towards a unique asymptotic asynchronous steady state of growth, where the overall number of cells increases exponentially, but the percentage of cells in each age and phase is constant [7]. In the practice, the steady state of growth represents a reasonable approximation of the common exponential phase of growth in cell cultures (but also *in vivo*, as far as environmental conditions remain constant), where cell cycle distributions are time-independent. By starting an experiment when the cell population is in this steady state, as it is usually done, the initial cell distribution was determined by a preliminary desynchronization run for each set of parameters' values.

In this way the models of steady state growth of BxPC-3 and Capan-1 were derived with the following parameters values: $\overline{T}_{G1}$ 8.6 h; $\overline{T}_{S}$ 11.0 h; $\overline{T}_{G2M}$ 4.7 h; CV_{G1} 85%; CV_S and CV_{G2M} 30% for BxPC-3 and $\overline{T}_{G1}$ 10.3 h; $\overline{T}_{S}$ 11.0 h; $\overline{T}_{G2M}$ 5.0 h; CV_{G1}, CV_S and CV_{G2M} 30% for Capan-1. These models predict contemporaneously cell cycle percentages and overall number of cells in each generation (Figure 1 – control) and the distribution of intermitotic times (Supplementary Figure 1) measured in untreated control cells of the two cell lines during the exponential phase of growth.

The computer program in use would enable to build models simulating cell cycling with higher complexity, e.g. introducing quiescent cells and loss and simulating a progressive approach to confluence of the cell population [5], but these effects were negligible and the study of these details was out of the aim of the present work.

Modeling response to treatment

The effects of treatment were described by parameters measuring delays, blocks and killing of cells in each phase. This is reflected in the structure of the modeling framework *in silico*, including modules for G_1, S and G_2M checkpoint responses in generations 0, 1, 2, etc., superimposing and modifying the flow of the cells through the cycle.

Modules render complex biological phenomena, such as the operation of a checkpoint in a specific cell cycle phase, with the minimal choice of parameters so that the main antiproliferative effects (block, block's recovery or death) occurring in that phase can be quantified, as previously described with full mathematical details [5].

Briefly, to test drug or radiation effects in a particular phase, the researcher can choose among different types of modules, rendering checkpoint activity with increasing levels of complexity. At the lowest level (type I), the effect is simply a delay of the progression in the phase where it is located, lengthening the phase. The balance equations inside a phase were modified by a "delay" parameter (Del) as follows:

$$N_{ph,genh}(a_{i+1}, t+\Delta) = (1-Del_{ph})\,(1-\beta_{ph}(a_i))\, N_{ph,genh}(a_i,t) + Del_{ph}\,(1-\beta_{ph}(a_{i+1}))\, N_{ph,genh}(a_{i+1},t)$$

meaning that a fraction $(1-Del_{ph})$ of cells progresses in the cell cycle while a fraction Del_{ph} remains in the same age compartment at each time step. Analogous straightforward modifications apply to other equations.

The associated "delay" parameter in S phase is equivalent to the fractional reduction of the average DNA replication rate, providing a quantitative measure of the inhibition of DNA synthesis, net of other confounding factors (like the overall number of cells or the number of dying cells) concurring in measures obtained with other tests. Cell death was regulated by a second, independent, "death rate" parameter (DR), simply representing the fraction of cells in the phase that die at each time step. Thus equations become:

$$N_{ph,genh}(a_{i+1},t+\Delta) = (1-DR_{ph})\,[(1-Del_{ph})\,(1-\beta_{ph}(a_i))\, N_{ph,genh}(a_i, t) + Del_{ph}(1-\beta_{ph}(a_{i+1}))\, N_{ph,genh}(a_{i+1},t)]$$

being $(1-DR_{ph})$ the fraction of cells surviving, while the fraction DR_{ph} is lost.

A type II module acts by arresting permanently a fraction of the cells transiting to the next phase in a specific compartment of blocked cells ("block probability" parameter, pBL_{ph}). Cell death is measured by a second, independent parameter – "death rate in block" ($DRBL_{ph}$) – representing the fraction of cells in the blocked-cell compartment that die at each time step. We used type I or II modules to emulate G_1 and G_2M checkpoint activities, while only type I was considered appropriate to describe the activity of S-phase checkpoint, resulting in a delay or complete inhibition of DNA replication and not in an arrest at a specific point within the S phase as assumed by type II. New equations introducing the compartments of blocked cells (B_{G1} and B_{G2M}) were added, while corresponding changes were applied to the first compartment in the subsequent phases, reached only by the fraction of non-blocked cells:

$$B_{G1,genh}(t+\Delta) = (1-DRBL_{G1})BG_{1,genh}(t) + pBL_{G1}\Sigma_i\beta_{G1}(a_i)\, N_{G1,genh}(a_i,t)$$

$$N_{S,genh}(a_1,t+\Delta) = (1-pBL_{G1})\, \Sigma_i\beta_{G1}(a_i)N_{G1,genh}(a_i,t)$$

$$B_{G2M,genh}(t+\Delta) = (1-DRBL_{G2M})\, B_{G2M,genh}(t) + pBL_{G2M}\, \Sigma_i\beta_{G2M}(a_i)\, N_{G2M,gen(h-1)}(a_i,t)$$

$$N_{G1,genh}(a_1,t+\Delta) = 2\,(1-pBL_{G2M})\Sigma_i\beta_{G2M}(a_i)\, N_{G2M,gen(h-1)}(a_i,t)$$

Type III aims at rendering a possible resumption of blocked cells in cycle, adding a new parameter describing recycling – "recycling rate" (Rec_{ph}), the fraction of cells in the blocked-cell compartment that resume cycling, entering the next phase – to the type II module. Further complexity can be introduced as desired, including, in addition to phase and generation dependence, a time-dependence for the onset or disappearance of one or more of the effects (block, recycling or death). The previous equations are accordingly modified:

$B_{G1,genh}(t+\Delta) = (1\text{-}RecG1)\ (1\text{-}DRBL_{G1})B_{G1,genh}(t) + pBL_{G1}\Sigma_i\beta_{G1}(a_i)N_{G1,genh}(a_i,t)$

$N_{S,genh}(a_1,t+\Delta) = Rec_{G1}(1\text{-}DRBL_{G1})B_{G1,genh}(t) + (1\text{-}pBL_{G1})\ \Sigma_i\beta_{G1}(a_i)N_{G1,genh}(a_i,t)$

$B_{G2M,genh}(t+\Delta) = (1\text{-}Rec_{G2M})\ (1\text{-}DRBL_{G2M})B_{G2M,genh}(t) + pBL_{G2M}\Sigma_i\beta_{G2M}(a_i)N_{G2M,gen}(h\text{-}1)(a_i,t)$

$N_{G1,genh}(a_1,t+\Delta)=$

$2\ [Rec_{G2M}\ (1\text{-}DRBL_{G2M})B_{G2M,genh}(t) + (1\text{-}pBL_{G2M})\ \Sigma_i\beta_{G2M}(a_i)\ N_{G2M,gen}(h\text{-}1)(a_i,t)]$

Notice that parameters of each module are probabilities (e.g. "block probability") or rates (e.g. "death rate") of a specific event in the phase and generation where the checkpoint is located, used in a first order approximation to determine the fraction of cells blocked or dead within the specific cohorts of cells arriving at the checkpoints. This enabled us to introduce the heterogeneity of the response to treatment in the model, avoiding overfitting by considering the rich data set of the time-course of flow cytometric and time-lapse data.

Building erlotinib and gemcitabine models

In a preliminary screening of biologically sound models we initially tested models including only G_1 delay in the case of erlotinib and S delay in the case of gemcitabine, optimizing parameter values, but these models were unable to predict the experimental time courses. The final models were reached by progressively increasing the number of parameters until simulation of the time course of cell cycling satisfactorily described the data for all drug concentrations and experimental platforms, avoiding over-fitting by the use of the likelihood ratio test.

The final gemcitabine model included the following modules:

1) S-phase module type I in generations 0 and 1, with time dependence of the Del_S parameter. Maximum reduction of the DNA synthesis rate (dependent on drug concentration) was reached immediately (i.e. within 0.5 h) when the drug was added and persisted several hours after drug removal. A sharply decreasing Hill function (sigmoidicity -10), with half-time (time to reach half the maximum value, or half the difference between maximum and minimum when minimum is not zero) of 18 h (generation 0) or 21 h (generation 1) was suitable in all treatment groups of both cell lines to describe the time-dependence of the S-phase delay parameter. At higher gemcitabine concentrations data were incompatible with complete recovery of DNA synthesis and a minimum long-term delay was maintained through generations 0 and 1. S-phase death rate was observable in BxPC-3 only after 48 h (generation 0) or 72 h (generation 1) and was described by an increasing Hill function (sigmoidicity 10) with half-time 48 h or 72 h.

2) G_1 phase module type I in generation 0, with time dependence of Del_{G1} parameter in generation 0. Time-dependence was described by a Hill function (sigmoidicity -10) with half-time depending on drug treatment (BxPC-3: 6 h, 15 h and 27 h for 20, 40 and 120 nM gemcitabine; Capan-1: 15 h for 100 nM; at 30 nM no G_1 delay was detected).

3) G_2M phase module type II in generation 0, with time dependence of pBl_{G2M} parameter. Time-dependence was described by an increasing Hill function (sigmoidicity 10), initially zero, with half-maximum at 12 h (BxPC-3) or 6 h (Capan-1), meaning that this effect did not involve cells initially in G_2M (which all exit before that time) but only cells reaching G_2M after recovery of the DNA synthesis rate in the previous S phase.

Thus seven variable parameters were optimized in each gemcitabine treatment group: maximum S delay (generations 0 and 1), long-term S delay in generation 0, long-term S delay in generation 1, S-phase death rate in generation 0, S-phase death rate in generation 1, maximum G_1 delay (generation 0), maximum G_2M delay (generation 0).

In the erlotinb model we separated the response in the periods during (0–48 h) and after (> 48 h) treatment, applying distinct modules in the two intervals as follows:

1) G_1 phase module type II in generation 0, with time dependence of pBl_{G1} during treatment. The onset of the effect was not immediate after drug addition: time-dependence was described by an increasing Hill function (sigmoidicity 1), initially zero, with half-maximum at 6 h.

2) G_1 phase module type II in generation 1 during treatment. The model cannot detect short-term time dependence for the onset of this effect, as a negligible amount of cells has time to complete G_1 in generation 1 in the first 6 h. Thus pBl_{G1} was kept time-independent.

3) S-phase module type I in generation 0, with time dependence of Del_S (sigmoidicity 1, half-maximum at 6 h) during treatment. The S-phase death rate was assumed constant and equal in generations 0 and 1, as the data were insensitive to more detailed, time-dependent models.

4) S-phase module type I in generations 1 and 2 during treatment. Because the data were not sensitive to different values in these generations, a unique Del_S parameter was used for both.

5) G_2M phase module type II in generation 0, with time dependence of pBl_{G2M} (sigmoidicity 1, half-maximum at 6 h) during treatment.

6) G_1 phase module type III in generations 0 and 1 after treatment (mainly). Although no more cells were blocked in G_1 after treatment, previously blocked cells exited the block with a recycling rate following an increasing Hill function with half-maximum at 48 h and sigmoidicity 10, meaning that the phenomenon actually started before the end of treatment. A unique pBl_{G1} parameter was used for both generations.

7) S-phase module type I in generations 1 and 2 after treatment. Because the data were not sensitive to different values in these generations, a unique Del_S parameter was used for both.

8) G_2M phase module type I in generations 1 and 2 after treatment. Because the data were not sensitive to different values in these generations, a unique pBl_{G2M} parameter was used for both.

The following nine parameters were optimized in each erlotinib concentration, during treatment: maximum G_1 block probability in generation 0, G_1 block probability in generation 1, maximum S delay in generation 0, S-phase death rate in generations 0 and 1, S delay in generations 1 and 2, maximum G_2M block probability in generation 0; and after treatment: maximum G_1 recycling rate in generations 0 and 1, S delay in generations 1 and 2, G_2M delay in generations 1 and 2.

In Capan-1, with the concentrations used in this study, the data were explained with a subset of six parameters, setting the following parameters to zero: S-phase death rate in generations 0 and 1, S delay in generations 1 and 2 and G_2M delay in generation 1 and 2.

Optimization and uncertainty analysis

Models were optimized by non-linear fitting of data for individual doses, simulating a complete time course with 0.5 h time step at each tentative set of parameters' values. The objective function to be minimized during the optimization was the overall negative log-likelihood (-log(L)), obtained by summing the contributions of all experimental procedures and assuming normally distributed errors with variance typical of each procedure [5].

We estimated the uncertainty of the best-fit parameters, calculating likelihood-based confidence intervals, giving the range for each parameter within which the fit remained not significantly worse than that obtained with the best-fit parameters, according to a likelihood ratio test. We took into account every parameter in the best scenario reproducing the experimental data and changed them, one by one. Likelihood-based 95% confidence intervals for each parameter were obtained by raising or lowering its value until log(L) was reduced from its best-fit value log(Lbest) to $\log(L) = \log(Lbest) - \chi^2_{0.05,1}/2$ [8].

REFERENCES

1. Ubezio P. Cell cycle simulation for flow cytometry. Comput Methods Programs Biomed. 1990; 31:255–266.
2. Montalenti F, Sena G, Cappella P, Ubezio P. Simulating cancer-cell kinetics after drug treatment: application to cisplatin on ovarian carcinoma. Physical Review E 1999; 57:5877–5887.
3. Lupi M, Matera G, Branduardi D, D'Incalci M, Ubezio P. Cytostatic and cytotoxic effects of topotecan decoded by a novel mathematical simulation approach. Cancer Res. 2004; 64:2825–2832.
4. Ubezio P, Falcetta F, Lupi M. Challenges in the integration of flow cytometry and time-lapse live cell imaging data using a cell proliferation model. In: D'Onofrio A, Cerrai P and Gandolfi A, eds. New Challenges for Cancer Systems Biomedicine. (Italia: SIMAI Springer Series, Springer-Verlag). 2012; 377–398.
5. Falcetta F, Lupi M, Colombo V, Ubezio P. Dynamic rendering of the heterogeneous cell response to anticancer treatments. PLoS Comput Biol. 2013; 9:e1003293.
6. Sisken JE, Morasca L. Intrapopulation Kinetics of the Mitotic Cycle. J Cell Biol. 1965; 25:179–189.
7. Watanabe I and Okada S. Effects of temperature on growth rate of cultured mammalian cells (L5178Y). J Cell Biol. 1967; 32:309–323.
8. Aitkin M, Anderson D, Francis B, Hinde J. Statistical Modelling in GLIM. (Oxford: Oxford University Press). 1989.

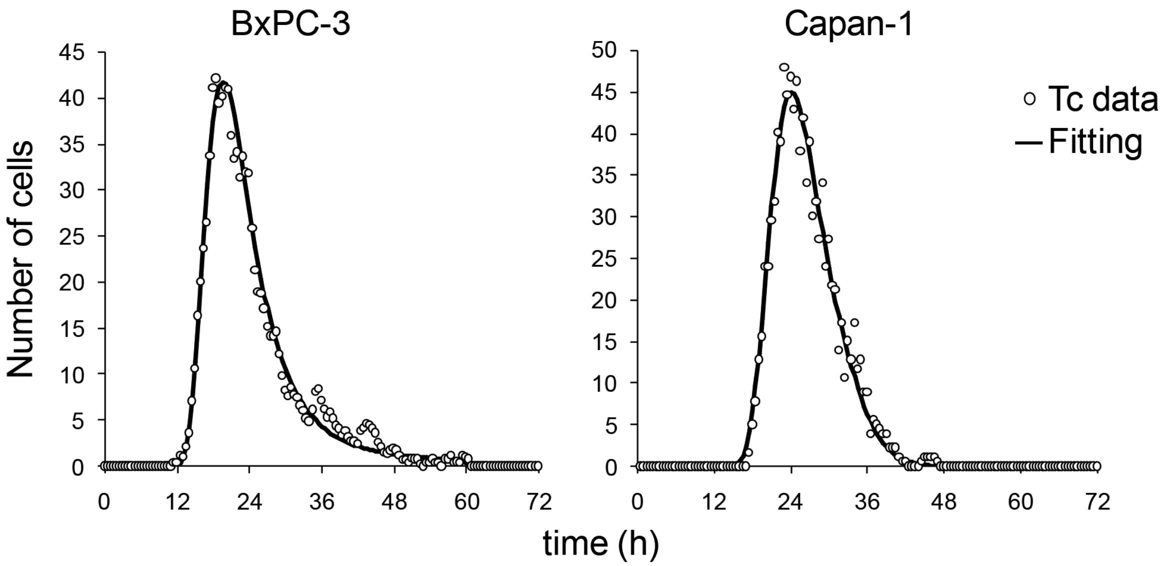

Supplementary Figure S1: Fitting of intermitotic time distributions. Intermitotic time distribution (Tc) of BxPC-3 and Capan-1 untreated cells obtained by time lapse analysis (open circle). The continuous line represents the fitting obtained by the computer simulation, described in Supplementary Methods.

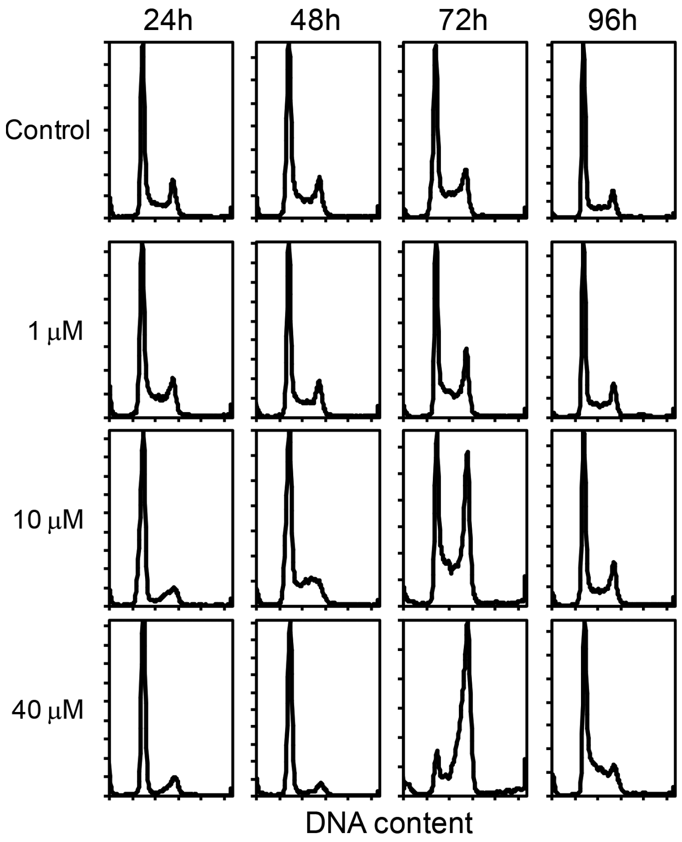

Supplementary Figure S2: Flow cytometric analysis of DNA content in BxPC-3 cells exposed to different concentrations of erlotinib for 48 h.

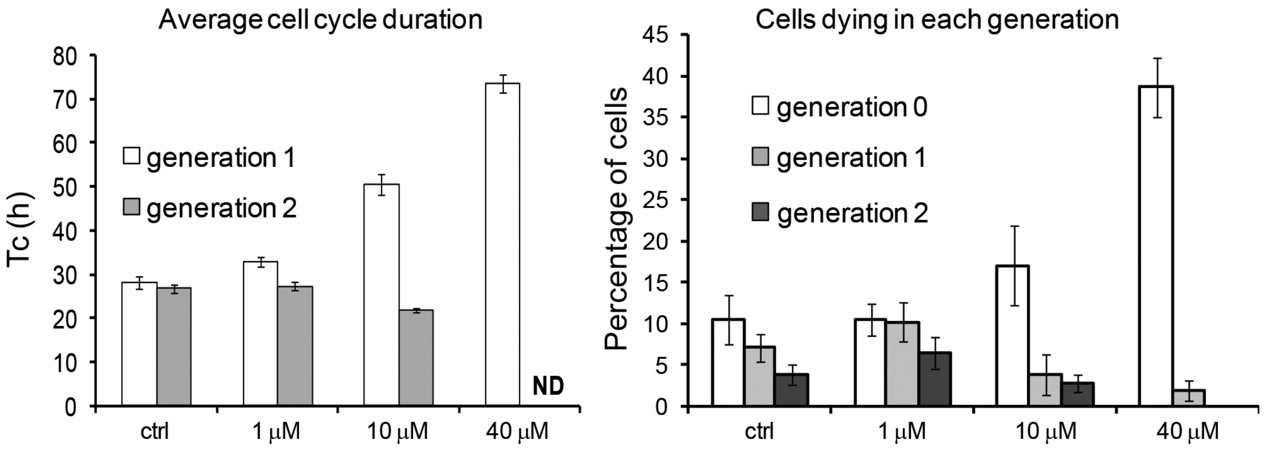

Supplementary Figure S3: Average cell cycle durations and percentages of cells dying in each generation derived from time-lapse experiments during and after treatment of BxPC-3 cells with different concentrations of erlotinib. Respectively 271, 138, 110 and 165 lineages were analyzed for control, 1, 10 and 40 μM erlotinib. Columns and error bars represent the mean and standard deviation of experimental data of five replicate culture wells.

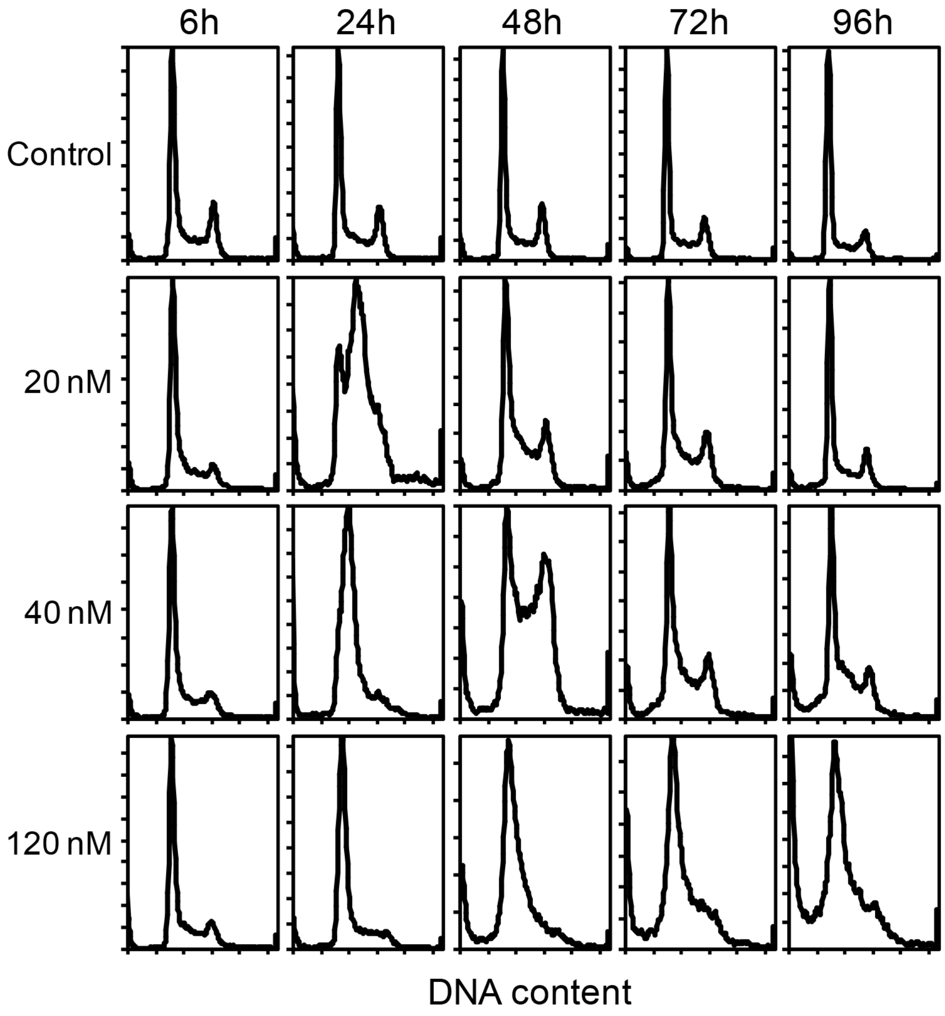

Supplementary Figure S4: Flow cytometric analysis of DNA content in BxPC-3 cells exposed to different concentrations of gemcitabine for 6 h.

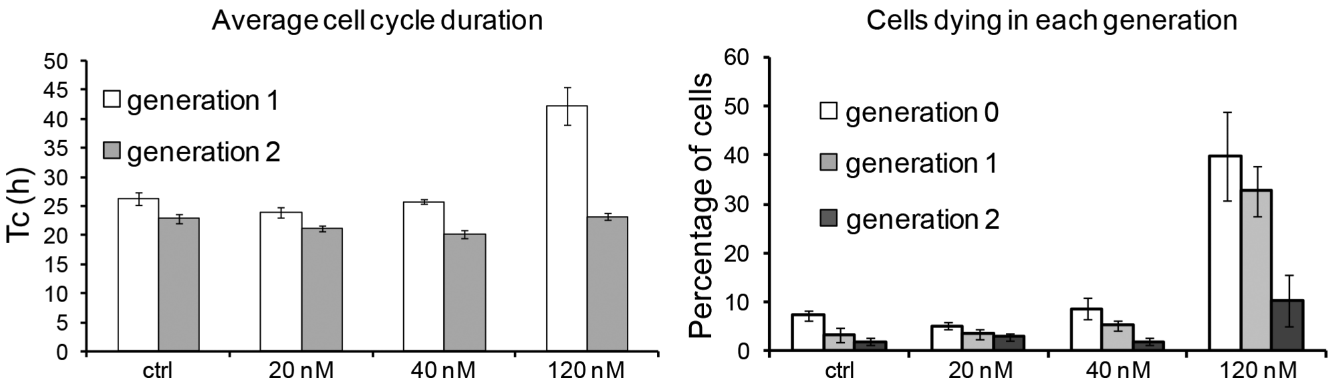

Supplementary Figure S5: Average cell cycle durations and percentages of cells dying in each generation derived from time-lapse experiments performed during and after treatment of BxPC-3 cells with different concentrations of gemcitabine. Respectively 178, 198, 226 and 110 lineages were analyzed for control, 20, 40 and 120 nM gemcitabine. Columns and error bars represent the mean and standard deviation of experimental data of five replicate culture wells.

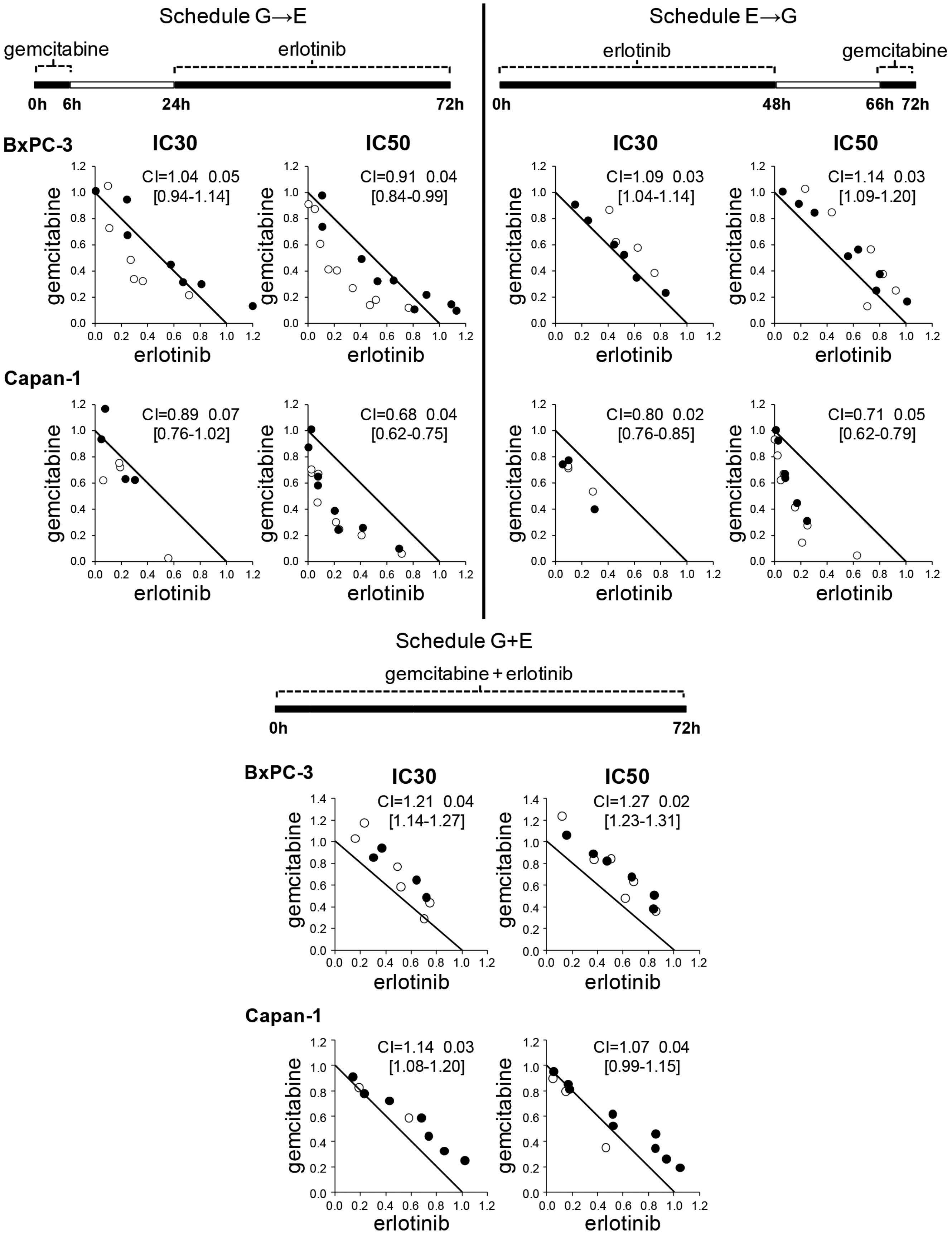

Supplementary Figure S6: Isobologram analysis of sequential and simultaneous treatment with erlotinib and gemcitabine. Isobolograms of erlotinib and gemcitabine combinations in BxPC-3 and Capan-1 cells at IC30 and IC50 with the corresponding combination indexes (CI) and confidence intervals. Abscissa: erlotinib concentration, as a fraction of erlotinib IC30 and IC50. Ordinate: gemcitabine concentration, as a fraction of gemcitabine IC30 and IC50. The additivity line separates the antagonistic (upper) from the synergistic (lower) region. Empty and filled circles represent the results of two independent experiments.

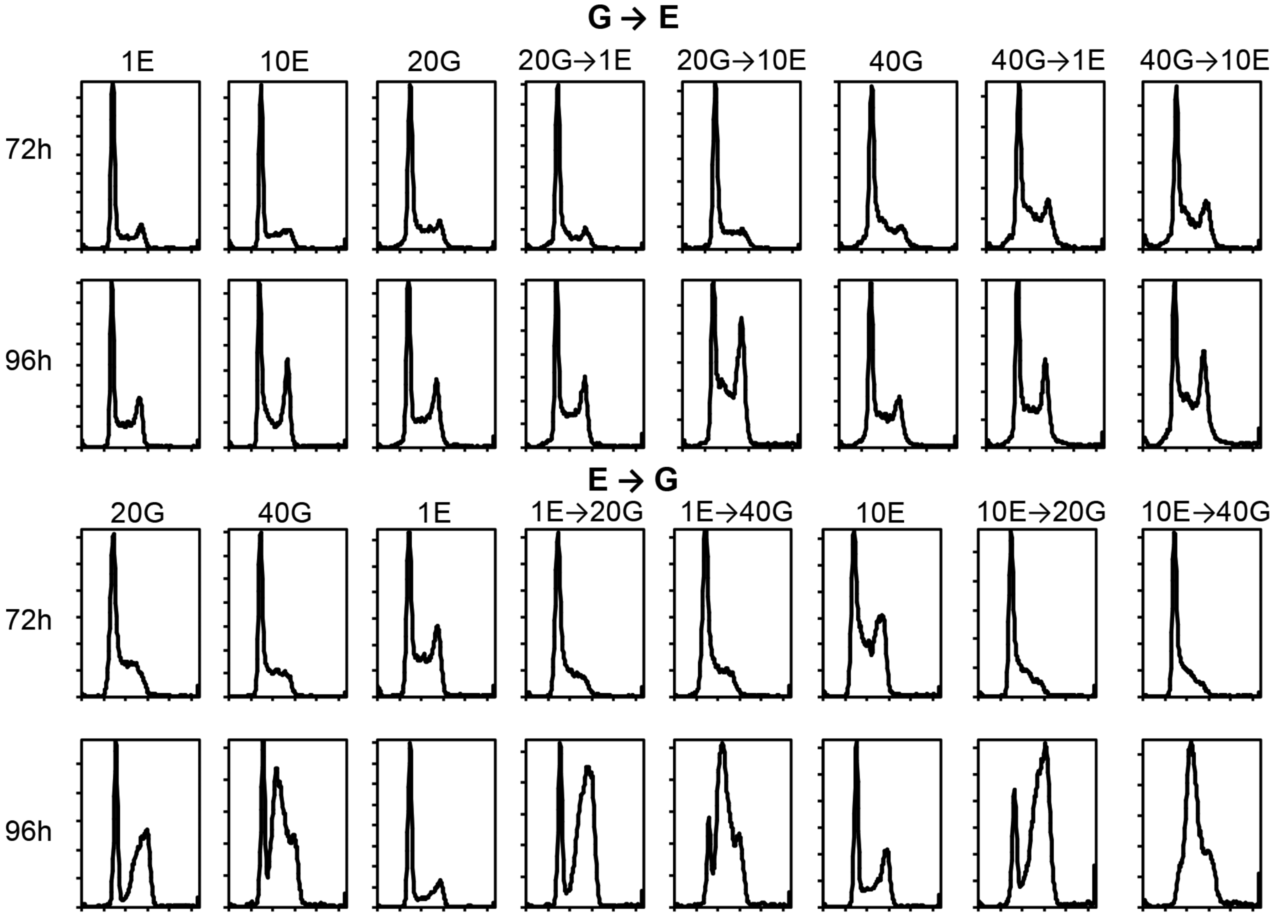

Supplementary Figure S7: Flow cytometric analysis of DNA content in BxPC-3 cells treated with the G→E and E→G sequences. Cells were fixed for FC analysis at the end of the second treatment (72 h) and 24 h after drug washout (96 h). Treatments: 1 μM erlotinib (1E), 10 μM erlotinib (10E), 20 nM gemcitabine (20G), 40 nM gemcitabine (40G). In the G→E sequence, cell cycle distributions at 96 h showed an increase of S-phase cells in gemcitabine pre-treated samples, indicating the different kinetics of the cells exiting the G_1 block after erlotinib. In the opposite sequence, DNA distributions mostly reflected the effect of gemcitabine, with a slightly larger lack of G_2M cells in samples pre-treated with erlotinib at 72 h. At 96 h we observed the same wave of synchrony as the single-drug samples, but with a smaller proportion of G_1 cells, suggesting that cells could recover more efficiently from the G_1 block.

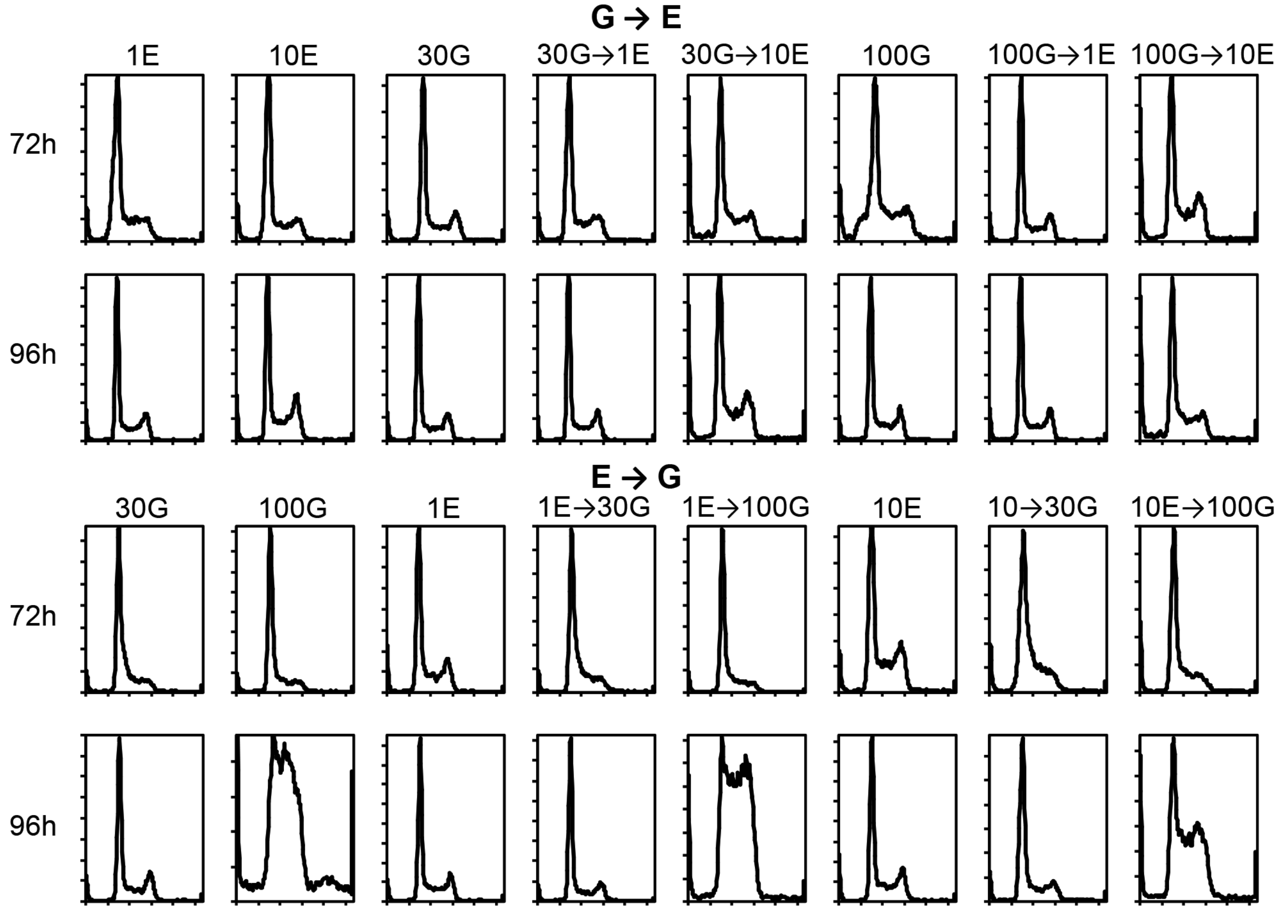

Supplementary Figure S8: Flow cytometric analysis of DNA content in Capan-1 cells treated with the G→E and E→G sequences. Cells were fixed for FC analysis at the end of the second treatment (72 h) and 24 h after drug washout (96 h). Treatments: 1 µM erlotinib (1E), 10 µM erlotinib (10E), 30 nM gemcitabine (30G), 100 nM gemcitabine (100G). In the G→E sequence, both single and combined treatments caused only very slight cell cycle perturbation. In the opposite sequence, the wave of synchrony traversing S phase after gemcitabine was clearly observed at 96 h only in the samples treated with 100 nM. Erlotinib pre-treatment (especially 10 µM) reduced this effect.
